# Exploring Subjective Well-Being in Human-Machine Interaction: Protocol for a Mixed Methods, Cross-Sectional Analysis in Manufacturing 5.0

**DOI:** 10.2196/73896

**Published:** 2025-11-14

**Authors:** Giulia Bassi, Valeria Orso, Silvia Salcuni, Luciano Gamberini

**Affiliations:** 1 Department of Developmental Psychology and Socialization University of Padova Padova Italy; 2 Human-Inspired Technology Research Center University of Padova Padova Italy; 3 Department of General Psychology University of Padova Padova Italy

**Keywords:** subjective well-being, operators, manufacturing 5.0, human-machine interaction, protocol study

## Abstract

**Background:**

Human-machine interaction (HMI) has gained significant attention in the context of advanced production technologies, especially concerning trust and acceptance. However, the investigation of the subjective well-being of operators working with these technologies in manufacturing companies has been largely overlooked. Moreover, previous research mostly relied on a single data-collection method, either quantitative or qualitative, thereby failing to capture a rich picture of their cognitive and affective states.

**Objective:**

This cross-sectional study protocol aimed to fill that gap by examining operators’ subjective well-being and workplace dynamics, including fluency in HMI, negative attitudes toward technologies, and social relationships among coworkers in manufacturing companies.

**Methods:**

We adopt a mixed methods approach, incorporating both quantitative and qualitative data collection techniques. Quantitative data will be gathered via a digital survey containing self-report questionnaires. A path analysis will be performed to explore the multiple mediating roles of fluency in HMI and negative attitudes toward such technologies between cognitive and affective well-being. We further qualitatively investigate the operators’ lived experience in HMI using semistructured audio-recorded interviews. A thematic analysis relying on text-mining techniques will then be conducted to explore operators’ textual data.

**Results:**

We quantitatively expect that fluency in HMI may act as a protective factor for operators’ affective well-being, while negative attitudes toward advanced production technologies may contribute to the development or worsening of operators’ psychological distress. From a qualitative perspective, we intend to seamlessly merge quantitative insights to create a more comprehensive and well-grounded analysis. Moreover, the integrated interpretation of both the quantitative and qualitative data collected will generate a consensus report, which will aim to serve as a practical framework for guiding workplace policies and training programs meant to foster subjective well-being and effective HMI. At the time of publication, we have collected data from 12 participants and scheduled a further data collection session.

**Conclusions:**

Embracing one of the fundamental pillars of Industry 5.0, human-centricity, by detecting potential psychological issues early, organizations can create a workplace that prioritizes the well-being of operators. Early recognition and prevention are crucial to promoting operators’ mental well-being involved in HMI.

**International Registered Report Identifier (IRRID):**

DERR1-10.2196/73896

## Introduction

### Background

In the last decades, the world of work has undergone radical changes across various sectors thanks to the rapid advancements of technological innovations. Technologies, such as the Internet of Things and cloud computing, have revolutionized how work is performed. In particular, according to the vision framed by the paradigm of Industry 4.0, the automation of the production processes would have significantly relieved workers from the heaviest and most tiring tasks [1]. However, the strong technology-driven focus of Industry 4.0 soon became evident, along with the limited accomplishment of the initial resolutions [2]. Indeed, the research that was carried out in this context sparsely focused on human factors [3]. According to a recent document published by the European Union [4,5], Industry 5.0 builds upon Industry 4.0 and is based on 3 founding pillars: human-centricity, sustainability, and resilience. Importantly, this shift reflects the recognition of the need to bring workers to the forefront of the innovation process, thereby designing and implementing technologies that meet their skills and needs. The emphasis on workers is not limited to providing them with the necessary competencies in using and interacting with novel advanced production technologies. It further includes a holistic approach that prioritizes their physical and subjective well-being. Subjective well-being, in particular, is the central focus of this study, conceptualized along 2 main dimensions, which are cognitive well-being (eg, satisfaction with life, job satisfaction, and work–home balance) and affective well-being (eg, stress, anxiety, and depression symptoms). While previous mixed methods studies in adjacent fields (eg, human-technology interaction) have addressed aspects such as technology acceptance or usability, they have rarely adopted an integrated framework that explicitly combines operators’ cognitive and affective well-being in an ecological context [6,7]. In particular, in the framework of Industry 5.0, several studies have investigated specific aspects related to the impact of novel technology on the interaction between human and technology itself, for example, technology acceptance [8], trust [9], and best practices for introducing this technology [10]. The quality of this interaction often depends on fluency, a construct that captures the smoothness and synchronization, and coordination between human and machine actions during shared tasks and encompasses different dimensions [11]. Higher levels of fluency are associated with increased trust in machines, particularly robots, improved teamwork, and reduced cognitive workload, thereby providing a more positive experience for workers [12]. Conversely, negative attitudes toward machine systems, including both emotional responses (eg, discomfort, fear) and social perceptions (eg, lack of perceived peer or organizational support), can act as significant barriers to successful human-machine interaction (HMI) [13]. These attitudes are often shaped by workers’ concerns about job security, lack of familiarity with technology, or perceptions of machines as competitors rather than collaborators [14,15]. Fluency in HMI and negative attitudes are positioned in this study as key mediating factors that may influence how cognitive well-being translates into affective well-being outcomes. Addressing these emotional and social dynamics is crucial for creating a balanced and supportive work environment where advanced production technologies can be effectively integrated.

To conceptually frame the relationship between work-related psychological outcomes and advanced production technologies, we draw on the Job Demands-Resources (JD-R) model [16]. According to this model, workplace factors can be broadly classified into job demands (ie, aspects that require sustained effort and are associated with psychological or physiological costs) and job resources (ie, aspects that support goal achievement, reduce job demands, or stimulate personal growth). In technologically intensive environments, advanced production technologies may play a dual function. Indeed, they may act as job resources by increasing task fluency, reducing physical and cognitive workload, and improving precision; but also, as job demands, when they introduce complexity, increase mental load, or elicit technostress, potentially due to lack of familiarity or negative attitudes. Our dual focus on how both cognitive and affective well-being are shaped by workers’ interaction with advanced equipment is attuned to the ambivalent role of technology illustrated by the JD-R model [16].

In addition, a growing body of research has addressed the potential threat that novel technologies can represent for workers, because of the possibility of job loss [13], and consequently has tried to identify the factors that can lead to their smooth introduction and integration [14]. However, the role played by operators’ affective and cognitive well-being has been largely overlooked by previous research, especially in the manufacturing sector. Similarly, the analysis of how the relationship between the worker and advanced production technologies develops over time, and how this may impact the subjective well-being and the social dynamics within the organization, has received little attention [15,17]. Indeed, previous research addressing the manufacturing industry mainly focused on the health risks related to physical interaction with technology [18], thereby failing to explore the psychosocial aspects implied and emerged from such interaction, with very few exceptions [18-21]. Moreover, in this novel paradigm, the home-work balance represents a crucial element [22,23] because it intertwines organizational and intrapersonal factors that can influence both affective and cognitive well-being. Recognizing and harmonizing these aspects is pivotal since they can enhance workers’ job satisfaction and productivity [24], as well as being able to promote satisfaction with life and a healthier and more sustainable work environment.

A further key aspect to consider is job satisfaction, which is defined as “a pleasurable or positive emotional state resulting from the appraisal of one’s job or job experiences” [24]. It encompasses a variety of dimensions that contribute to an individual’s overall contentment with their professional role. One significant dimension is the home-work interface, which plays a pivotal role in determining job satisfaction by influencing the interplay between professional responsibilities and personal life [25]. This balance not only can affect employees’ subjective well-being and productivity but also contributes to long-term career satisfaction and organizational commitment [24,25].

**Figure 1:**
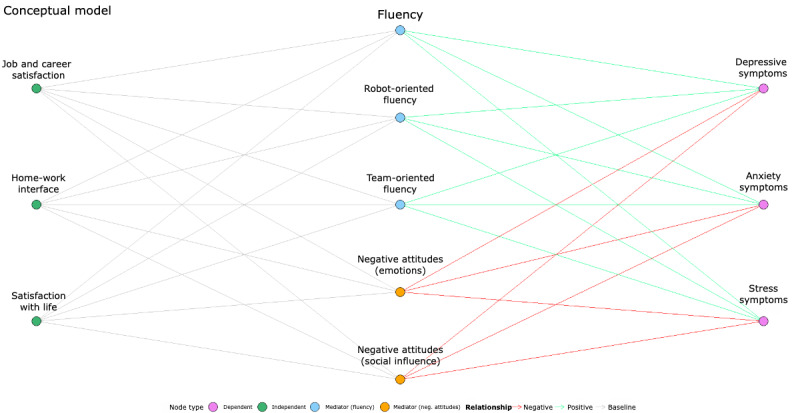
The conceptual model of a path analysis investigating the role of fluency in HMI and negative attitudes towards advanced production technologies between cognitive and affective well-being.

From the picture outlined above, it clearly emerges a critical gap in the current literature regarding the impact of advanced production technologies on the holistic subjective well-being of operators, particularly in manufacturing contexts. More specifically, while factors such as acceptance and trust have been addressed, there is a lack of integrated models that place operators’ cognitive and affective well-being at the forefront. This missing focus represents a significant limitation of previous research. Addressing this knowledge gap is crucial to developing human-centered interventions aligned with Industry 5.0 principles. Therefore, by adopting a structured conceptual hierarchy, this study conceptualizes indicators of cognitive well-being as independent variables, with affective well-being as the primary outcome (dependent variable). Fluency and negative attitudes toward advanced machines are positioned as key mediators within this framework, as illustrated in Figure 1. More specifically, we will focus on operators’ perceptions in interacting with advanced production technologies in order to investigate their affective well-being, such as anxiety, depression, and stress symptoms, along with their cognitive well-being, including satisfaction with life and the work-related quality of life (WRQoL) dimensions (ie, job and career satisfaction and home-work interface) in the context of HMI in manufacturing 5.0.

### Literature Review

#### State of the Art of Research Investigating Operators’ Subjective Well-Being in HMI Manufacturing Industries

The integration of new technologies in manufacturing industries not only revolutionizes production practices but also introduces complex dynamics that can have an impact on operators’ mental health [26]. Previous research conducted both with workers from manufacturing and other industrial sectors has extensively focused on the consequences of stress. Findings pointed out that work-related stress symptoms have been linked to various factors, including job insecurity, high physical and cognitive workloads, and work intensity [27-29]. While stress symptoms may be a short-term response to the demands of the workplace, long-term exposure can lead to chronic psychological problems, including depression symptoms [26]. Depression symptoms may profoundly alter the way individuals perceive and interact with their environment [27] and are likely to be further affected by the advent of automation and advanced production technologies in the workplace [12,30]. As such, technologies can increase feelings of alienation and job insecurity, reduce opportunities for meaningful interpersonal interactions, and impose rapid adaptation to constantly evolving systems. Consequently, these factors may amplify the sense of disconnection and the onset of stress and anxiety symptoms, highlighting the need for supportive interventions that consider the psychosocial impact of technological change [31-33].

Anxiety symptoms, on the other hand, although closely related to depression and stress symptoms, manifest differently and require independent consideration in both studies and interventions. Anxiety symptoms are often interpreted as being a by-product of the rapid change of advanced production technologies and the resulting demands in work environments, for example, high-intensity pace and constant need for digital skill updates [34,35]. Notably, anxiety symptoms may further burden the individual’s cognitive load, thereby creating a situation of overload. This is especially the case with anxiety symptoms triggered by the introduction of advanced production systems, and altogether they can substantially impair pivotal skills, such as decision making and problem solving [35].

While depression and anxiety are well-recognized mental health conditions with distinct profiles, their specific associations with HMI in advanced production environments remain poorly explored [36]. Previous studies have rarely investigated these symptoms as outcomes of cognitive well-being pathways mediated by fluency and negative attitudes, which represents a significant empirical gap.

Furthermore, a recent study found that high levels of fluency, which are related to the well-synchronized interplay between the human and the machine actions, are positively related to long-term teamwork, increased perception of one’s mental contribution, and higher trust in the machine, particularly robots [9,12,37]. Conversely, negative attitudes and emotional responses toward machines may play a significant role in the onset or increase of psychological distress. Additionally, the relationship between these negative perceptions and psychological symptoms remains underexplored in existing literature. Mindful of the above, it is imperative to thoroughly investigate these abovementioned psychological symptoms to better identify both protection and risk factors and to design purposeful intervention strategies for promoting and supporting operators’ subjective well-being and organizational effectiveness. Organizational effectiveness can be defined as the extent to which stakeholders’ goals, such as those of investors, employees, and the community, are achieved. It is enhanced when employees engage in behaviors that extend beyond their formal roles, commonly referred to as organizational citizenship behaviors. These behaviors are more likely to be exhibited by employees who experience high levels of job satisfaction [38,39].

In this regard, most of the studies investigating HMI, especially those conducted in the field, have relied solely on quantitative self-reported instruments, thereby highlighting the need to widen the methodological approach [10]. The few works investigating HMI using interviews provided rich descriptions of how the work environment, the tasks, and the social dynamics changed after the introduction, for instance, of a collaborative robot (cobot) on the shop floor [40-42], yet they overlooked the operators’ psychological state. However, it is important to note that most studies cited above had a more limited scope. Consistently, they have predominantly relied on cross-sectional quantitative surveys, often leaving behind the social dynamics and lived experiences of operators through qualitative or mixed methods approaches. Moreover, complex mediation models to understand the mechanisms linking cognitive well-being resources with affective well-being outcomes have been rarely adopted in previous research. Consequently, there is a need for research that combines quantitative rigor with qualitative depth to generate a more nuanced understanding of how advanced production technologies impact operators’ subjective well-being in practice. This study aims to address these gaps by integrating quantitative and qualitative methods within a single coherent framework through a triangulation strategy.

### Objective

#### Overview

The overall goal of this study is to deepen our understanding of operators’ subjective well-being while interacting with various advanced production technologies (eg, cobots, machine vision, and pick-to-light) in manufacturing companies, by subsequently formulating a consensus report aimed at improving workplace conditions. This consensus report will provide not only actionable guidance to enhance workplace policies and training programs but also serve as a foundational tool to promote sustainable and human-focused work environments, in line with Industry 5.0 principles.

We thus move away from a merely technology-oriented standpoint to a more holistic view that places operators, their subjective well-being, and their experiential interactions with advanced production technologies at the center. Therefore, in line with the principles of Industry 5.0, our investigation is grounded in a human-centric approach. Further enriching the depth of our study, we draw from both the fields of clinical and organizational psychology, emphasizing the importance of considering the operators’ perspectives of these technologies and their subjective well-being by adopting an interdisciplinary approach. More specifically, the purpose of this study is twofold. First, and more broadly, we aimed to explore through the administration of self-report questionnaires the multiple mediating roles of fluency (human-oriented fluency, machine-oriented fluency, and team-oriented fluency) and the negative attitudes toward these technologies. Specifically, we place cognitive and affective well-being at the center of our investigation, considering them as the main dimensions of interest.

To explore how these outcomes are shaped, we identify (refer to Table 1) (1) cognitive well-being indicators (ie, satisfaction with life, job and career satisfaction, home-work interface) as independent variables, (2) fluency in HMI and negative attitudes as key mediators, and (3) affective well-being outcomes (ie, stress, anxiety, and depression symptoms) as dependent variables.

Within this conceptual model, we hypothesized 2 distinct mediation mechanisms.

**Table 1:** Summary of the constructs investigated through the specific tool.

Constructs			Definition		Investigated with		
					Questionnaire		Semistructured interviews
**Affective well-being**							
	Depression symptoms	Feelings of persistent and frequent sadness, loss of interest or pleasure in activities, fatigue, and feelings of worthlessness or hopelessness. It can also involve physical and cognitive symptoms, such as changes in appetite, sleep disturbances, fatigue, and difficulty in concentrating.		✓			
	Anxiety symptoms	Excessive worry, fear, and heightened physiological arousal, such as restlessness, rapid heartbeat, and hypervigilance.		✓			
	Stress symptoms	Stress is a physiological or psychological response to internal or external stressors. Stress symptoms are a human response to cope with life’s challenges and threats. The presence of long-term stress symptoms and the way they are coped with can have a negative impact on subjective well-being, which can in turn impact on the organizational one.		✓			
**Cognitive well-being**							
	Home-work interface	The balance and harmony between home and work life.		✓			
	Job (and career) satisfaction	The positive attitude individuals have regarding their job and career advancement.		✓		✓	
	Satisfaction with life	Life satisfaction is related to the assessment of individuals’ quality of life.		✓			
**Human factors**							
	Physical and mental workload	The physical workload is the physical effort required by an operator in a manufacturing company. With the integration of robots, the physical workload can be modified. Mental workload refers to the cognitive and psychological demands placed on an operator.				✓	
	Team-oriented fluency in human-machine interaction	Well-synchronized interplay of human and machine actions.		✓		✓	
	Negative attitudes and emotions toward advanced production technologies	Individuals’ attitudes and emotions when interacting with advanced production technologies of different types.		✓			
	Trust	The dispositional attitude that a machine will support the achievement of the individual’s goals in a situation characterized by uncertainty and vulnerability.				✓	
	Acceptance	The beliefs that machines will be helpful for the individuals’ work and that they will not threaten their role in the workforce community.				✓	
	Usability	The extent to which working with the machine is effective, reasonably effortless, and satisfactory.				✓	
	Introduction of advanced production technologies and training	The modality that was adopted by the organization to introduce the advanced production technologies and provide training.				✓	
	Perceived risks and benefits	Exploration of which changes are deemed beneficial and which are perceived as a threat in HMI^a^ domains.				✓	
	Social relationships among coworkers	The extent to which the introduction of advanced production technologies has potentially changed the social dynamics among coworkers.				✓	

^a^HMI: human-machine interaction.

#### Higher Levels of Fluency

Higher levels of fluency (human-oriented, machine-oriented, and team-oriented) are hypothesized to positively and partially mediate the relationship between cognitive and affective well-being. Fluency is expected to act as a protective factor, facilitating smoother interactions with advanced production technologies and thereby reducing psychological distress (ie, anxiety, stress, and depression symptoms). As a mediating mechanism, fluency indirectly alleviates psychological distress by lowering cognitive workload and minimizing task-related frustrations inherent in complex HMI [38,39,42]. Partial mediation is proposed since evidence from HMI research indicates that while fluency reduces emotional strain, it cannot fully eliminate all sources of psychological distress [38]. For example, even operators with high levels of fluency may experience stress symptoms due to external factors, such as organizational change, time pressures, or conflicting demands, which directly have an impact on affective well-being [38].

#### Negative Attitudes Toward Advanced Production Technologies

Negative attitudes toward advanced production technologies (both emotional and social influence dimensions) are hypothesized to function as a complete mediator of the relationship within the tested model, acknowledging that other potential mediators might exist. This assumption is based on previous studies that observed that negative attitudes capture the emotional and social mechanisms that diminish well-being in technology-intensive environments [39].

Second, using semistructured audio-recorded interviews, we aim to conduct an in-depth qualitative analysis of operators’ perceptions of their subjective well-being and lived experiences within the context of HMI, considering different dimensions: introduction and training, human factors, cognitive and physical workload, trust, machine capabilities, acceptance, usability, social relationships among coworkers, and risks and benefits. For the extraction and understanding of pivotal textual data, we will use an advanced text-mining methodology [43,44].

The integration of both quantitative and qualitative data through a triangulation strategy will be adopted to enhance the validity and depth of the findings.

At the conclusion of the data collection and analysis, should specific areas of difficulty emerge, an optional psychoeducational intervention will be proposed to the organization. Such focus groups will aim to provide operators with practical tools to enhance their subjective well-being.

To our knowledge, this is the first cross-sectional study to comprehensively examine the relationship between the variables outlined above on operators using, interacting, and collaborating with advanced production technologies within manufacturing companies.

## Methods

### Overview

A checklist flow summarizing the study methodology is provided in Figure S1 in the Multimedia Appendix 1.

Following the call made by Meissner et al [10], this cross-sectional study will implement a mixed methods approach, incorporating both quantitative and qualitative data collection techniques. Both the quantitative and qualitative parts of the study have been conceived to be intimately related, yet independent. Participation in the semistructured interviews is voluntary, and as such, it is possible that some operators who complete the survey may choose not to participate in the interviews, ensuring flexibility. The semistructured interviews are designed to focus specifically on the operators’ perceptions of working and interacting with advanced production technologies, by further providing an opportunity for participants to share experiences that may not be fully captured in their survey responses.

More specifically, as regards the quantitative section, a digital survey containing the self-report questionnaires will be administered, relying on an online platform, namely Qualtrics. The duration of the digital survey will be approximately 20 minutes. Before filling in the questionnaire, the operators should read the study information and sign informed consent to participate in the study. The digital survey will start by asking (1) sociodemographic questions, such as age, sex, nationality, educational level, marital status, and presence or absence of offspring. Subsequently, (2) questions regarding the company in which the operators work will solicit information on their role, tenure, work shifts, and the specific advanced production technologies they work and engage with, as well as their tasks (ie, picking, packing, palletizing, welding, assembling items, handling, inspecting products for quality, disassembly, holding heavy weight, transporting goods, and other). Operators should then complete (3) a brief battery of standardized self-report questionnaires designed to assess their satisfaction with life, job, and career satisfaction, home-work interface, depression, anxiety, and stress symptoms, the negative attitudes toward advanced production technologies, and fluency in HMI.

The qualitative section will use semistructured audio-recorded interviews designed to last approximately 60 minutes. These semistructured interviews have the purpose of exploring the operators’ perceptions and lived experiences in working and interacting with advanced production technologies. In particular, the areas addressed will cover 9 dimensions related to the operators’ subjective well-being (ie, the introduction of technologies and training, human factors, physical and cognitive workload, trust, acceptance and usability, capabilities of machines, potential risks and benefits, and social relationships among coworkers).

As reported in the objective, this study further adopts a triangulation strategy, where qualitative data will be used to enrich and contextualize the quantitative results. This integration aims to provide a more comprehensive understanding of the psychological dynamics underlying operator interaction with advanced production technologies.

At the end of the data collection, psychoeducational interventions will be implemented through the means of the focus groups. Specifically, based on questionnaire outcomes related to cognitive and affective well-being, psychoeducational focus groups will be proposed to the company, and operators will be invited to participate on a voluntary basis. Each focus group will be carried out separately for each participating company, highlighting the unique organizational and contextual factors affecting operators within their specific workplace. The intervention will be moderated by 2 psychologists (a clinical and an organizational psychologist), addressing the potential psychological distress of operators. The focus groups are designed as part of the intervention strategy. While the qualitative section primarily aims to gather in-depth insights into operators’ lived experiences and perceptions through semistructured interviews, the focus groups serve a distinct purpose. They intend to provide psychoeducational support by potentially improving the operators’ overall well-being.

### Funding

The research is funded within the framework of the Ecosystem INEST-Interconnected Northeastern Innovation (PNRR) project and has already received approval from the ethics committee of the Human Inspired Technologies Interdepartmental Research Center of the University of Padua, Italy (2023_212R1). Notably, the content of the manuscript represents exclusively the Authors’ point of view; neither the European Union nor the European Commission should be considered accountable for its content.

### Study Population and Recruitment

#### Participants and Recruitment Overview

We will recruit operators working in different manufacturing companies located in Northeastern Italy, particularly those dealing with advanced production technologies.

To determine the necessary sample size required for pursuing the objective related to the quantitative section, a power analysis was conducted, using the semPower library [45] under the RStudio environment, assuming a root mean square error of approximation effect cut-off equal to 0.10 (medium-sized misspecification), a *P* value of .05, a power of 0.80, and *df*=52. The computation resulted in a minimum of 60 participants.

Notwithstanding this outcome, we acknowledge that the estimated sample size represents the minimum threshold required for adequate statistical power and we recognize that detecting individual indirect effects in mediation models often requires larger samples, particularly when assuming small-to-moderate path coefficients (eg, β=.20, .30). These estimates are consistent with effect sizes commonly reported in psychological models of workplace well-being and human-technology interaction [46]. Therefore, we aim to exceed the minimum threshold by expanding recruitment efforts across participating companies.

#### Inclusion and Exclusion Criteria

Given the purpose of this study, specific inclusion and exclusion criteria have been delineated. The criteria for participation are outlined as follows: (1) prospective participants should fall within the age range of 18 to 65 years, ensuring that the sample represents the working-age population within the manufacturing company; (2) being employed as shop floor operators or in a manufacturing company that has introduced advanced production technologies, thereby having first-hand experience with such technologies; (3) manufacturing companies in which advanced production technologies, such as cobots, pick-to-light systems, and machine vision technologies, have been introduced within the last 5 years. This criterion ensures that the study focuses on operators interacting with relatively recent implementations of advanced production technologies, reflecting the evolving practices of Industry 5.0. Moreover, and finally, (4) willingness to participate and provide informed consent. Participants will be excluded if (1) not directly engaged in the use of advanced production technologies and (2) diagnosed with cognitive impairments or any other conditions that might hinder their ability to understand, engage in, or provide feedback.

### Quantitative Data Collection: Self-Reported Questionnaires

All the self-report questionnaires are validated in Italy for adults’ respondents, except for the fluency in the HMI scale [11], for which we carried out a back-translation procedure. Moreover, all the below-mentioned instruments have demonstrated good psychometric properties.

#### Depression Symptoms

Depression symptoms will be measured through the Patient Health Questionnaire-9 [47], which is a brief self-reported unidimensional instrument that incorporates the *DSM-IV-TR* (*Diagnostic and Statistical Manual of Mental Disorders* [Fourth Edition, Text Revision]) [48]. The questionnaire was developed to assess and monitor the severity of depression symptoms in the previous 2 weeks through 9 items rated on a 4-point Likert scale, from 0 (“never”) to 3 (“almost every day”). A threshold score of 10 is indicative of clinically significant depression symptoms (scores 0-27). The Patient Health Questionnaire-9 stratifies depression severity into 5 severity categories: absent, subthreshold depression symptoms, mild depression symptoms, moderate depression symptoms, and major depression symptoms. Assessing depression symptoms, for example, persistent and frequent feelings of sadness, guilt, and worthlessness, is relevant as depression is often considered a proxy of the individual’s mental health [20]. Moreover, depression symptoms can alter how the individual perceives events, and they can result in long-term exposure to stress symptoms [26,30].

#### Anxiety Symptoms

Anxiety symptoms will be evaluated using the Generalized Anxiety Disorder-7 (GAD-7) scale [49], which is a brief self-reported unidimensional measure aimed at screening probable cases of GAD and assessing the severity in the previous 2 weeks. The GAD-7 comprises 7 items based on a 4-point Likert scale, from 0 (“never”) to 3 (“almost every day”). A score equal to 10 indicates relevant anxiety symptoms (score ranging between 0 and 21). The questionnaire included 3 categories of severity according to the DSM-IV-TR criteria: mild anxiety symptoms, moderate anxiety symptoms, and severe anxiety symptoms. The investigation of anxiety symptoms is motivated by the fact that they may be caused by the rapid change in production technologies and the consequent new demands in one’s job, leading to significant psychological distress [12].

#### Satisfaction With Life

Satisfaction with life scale [50] will be administered to assess operators’ global cognitive perception of their life satisfaction. The satisfaction with life scale is a 5-item self-report unidimensional questionnaire based on a 7-point Likert scale, from 1 (“strongly disagree”) to 7 (“strongly disagree”). Assessing the overall level of life satisfaction is relevant in this context because higher levels of life satisfaction are linked to better job performance [51]. Additionally, it was found that work-related events impact one’s level of satisfaction with life [52].

#### WRQoL

WRQoL [21] will be assessed through the work-related quality of life scale. The WRQoL is a multidimensional instrument characterized by 25 items plus an extra item, which investigates the overall level of WRQoL, rated on a 5-point Likert scale, from 1 (“strongly disagree”) to 5 (“strongly agree”). These items are divided into 7 factors (ie, control at work, home-work interface, job and career satisfaction, working conditions, employee engagement, and stress at work). This instrument presents the advantage, compared to other measures, of allowing researchers, organizational, and clinical professionals to assess WRQoL in both organizational and clinical settings [20].

In line with the purpose of this study, we will focus on the home-work interface, job and career satisfaction, and stress at work, further including the extra item to reflect an overall level of operators’ WRQoL. The choice to focus only on specific subscales is motivated by the role that those constructs play in the individual’s well-being. Indeed, advanced production technologies are known to foster unlimited connection, thereby threatening the separation between work and personal domains [53]. Specifically referring to human-cobot interaction, poor synchronization in the actions of human and robotic agents is related to lower levels of job satisfaction [54,55]. Finally, the introduction of new industrial systems, such as cobots, and the conversion of the workers’ role from operator to supervisor have led to a significant increase in their stress levels, which can negatively impact different human functions [26].

#### Fluency in HMI

The quality of HMI will be evaluated using the Fluency in Human-Robot Interaction Scale [11]. The Fluency in Human-Robot Interaction Scale is a multidimensional instrument characterized by 6 items rated on a 7-point Likert scale, from 1 (“strongly disagree”) to 7 (“strongly agree”). The scale is composed of 3 factors, namely human-oriented fluency, which evaluates the worker’s emotional states experienced during the interaction, robot-oriented fluency assesses the perception of the robot’s contribution during the collaboration, and team-oriented fluency refers to the team’s harmony. Including the assessment of fluency is grounded by the fact that a well-coordinated meshing of human and robots’ actions was found to positively influence long-term teamwork, increase the perception of one’s mental contribution, and foster higher levels of trust in the robot [11,51,52]. While this scale originally addressed the interaction between the worker and the robot, we extended its purpose to the broader context of HMI, thereby generally referring to advanced production technologies.

#### Negative Attitudes Toward Advanced Production Technologies

The negative attitudes toward advanced production technologies will be assessed using the Negative Attitude toward Robots Scale (NARS) [56]. The NARS is a multidimensional instrument designed to evaluate the negative attitudes that workers can experience in their interaction with robots. The NARS is composed of 17 items rated on a 5-point Likert scale ranging from 1 (“strongly disagree”) to 5 (“strongly agree”), encompassing three scales, (1) negative attitudes toward situations of interaction with robots, (2) negative attitudes toward the social influence of robots, and (3) negative attitudes toward emotions in interaction with robots. The inclusion of only these latter 2 subscales was driven by the need to align with the research objectives while ensuring a streamlined and efficient questionnaire completion process for respondents. Moreover, attitudes toward robots are worthy of investigation because they are affected by several factors, including expertise in the job, and it is also related to the level of trust in the machine systems [57,58].

#### Qualitative Data Collection: Audio-Recorded Semistructured Interviews

##### Qualitative Data Collection Overview

A semistructured interview protocol, called Exploration of Psychological Impacts in Collaborative Operator Settings (EPICOS) [59], combines open-ended questions with dichotomous and multiple-choice scales, creating a holistic understanding of the dynamics related to the HMI. Through EPICOS, we aim to highlight 9 key dimensions related to operators’ subjective well-being when working and interacting with advanced production technologies in the context of manufacturing 5.0. In particular, the 9 areas comprise several open-ended, dichotomous responses and Likert scale questions. The 9 areas that will be analyzed, along with their summarized questions, are reported below.

##### Introduction of Advanced Production Technologies and Their Training

This area of investigation refers to the modalities with which the introduction of advanced production technology is communicated to employees and the training they receive, which significantly contributes to how workers receive and interact with it [6].

##### Human Factors, Including Physical Ergonomics, and Tasks

Questions in this area explore how operators and advanced production technologies interact in Industry 5.0, and the organizational and clinical implications that their interaction has for the subjective well-being of the operators. It is well-known that the characteristics of the task allocation, the speed, and trajectory of the machine significantly impact the worker’s cognitive state [16,60].

##### Trust

This area investigates the workers’ attitudes and beliefs that the technological agent will help in accomplishing the task. The level of trust in technologies is key to ensuring successful HMI cooperation, and it also influences the use that workers make of technology [9,16,61].

##### Usability and Acceptance

These questions refer to factors related to the ease of interacting with the machine and the extent to which workers show a genuine willingness to work with it. These aspects are relevant as poor usability hinders a smooth and satisfactory usage of the technology, while low levels of acceptance hamper the very use and adoption of the technology itself [61].

##### Perceived Risks and Benefits

This area refers to the potential threats that workers associate with technology and the envisioned or experienced advantages related to it. More specifically, this area will encompass both the risks and benefits pertaining to physical interaction with technology, for example, safety issues, the wider perspective, and the role replacement. Attributing a certain level of risk to technology can negatively affect the acceptance of the technology itself [8].

##### Interpersonal Relationships Among Coworkers

This area of investigation is meant to explore whether and how the presence of advanced production technology has changed the relationships among coworkers. This area of investigation is relevant because social relationships at work significantly impact individuals’ well-being [10].

Given that the EPICOS protocol was specifically developed for this study, no formal validation or piloting was conducted. However, the interview guide was carefully reviewed by an interdisciplinary team of experts in organizational psychology, clinical psychology, and HMI to ensure content coherence, clarity of language, and contextual relevance for manufacturing settings. As a qualitative tool, EPICOS was designed to flexibly explore operator experiences across predefined psychosocial dimensions rather than to generate standardized quantitative outcomes.

### Statistical Analysis

Statistical analyses will be conducted using R custom code through the RStudio environment.

Descriptive statistics for both discrete (eg, frequencies) and continuous variables (eg, mean, SD, skewness, and kurtosis) will be run.

Bivariate Pearson *r* correlation coefficient will be deployed for evaluating the association between age, cognitive well-being (ie, job and career satisfaction, home-work interface, and satisfaction with life), affective well-being (ie, anxiety, depression, and stress symptoms), negative attitudes toward advanced production technologies, and fluency in HMI (*P*<.05). According to Cohen guidelines [62], correlation coefficients ranging between 0.10 and 0.29 indicate a “small effect size,” between 0.30 and 0.49 represent a “medium effect size,” and above 0.50 represent a “large effect size.”

A path analysis will be carried out to explore the direct and indirect associations between operators’ cognitive well-being as independent variables and affective well-being as the dependent variable. Central to this analysis is the investigation of the multiple mediating roles of fluency in HMI and the negative attitudes toward advanced production technologies, by highlighting the indirect effect pathways between the abovementioned variables. The strength and direction of these associations will be quantified using standardized regression coefficients (β), and the robustness of these estimates will be reported through CI.

A thematic analysis will be undertaken by relying on the text-mining technique, using the Quanteda package [63], to capture textual data within interview transcripts, thus highlighting emergent patterns and themes. The analysis will be carried out on the verbatim transcripts of the semistructured interviews by turning any numbers into letters and removing punctuation and stopwords. Subsequently, all semistructured interviews will be analyzed to explore the most frequent co-occurrences and digrams, based on a rule-of-thumb, in which words that will be repeated a minimum of three times will be considered co-occurrences as well as digrams [64]. The findings from this analysis will be cross-referenced with the quantitative results to ensure consistency and to identify areas of alignment or divergence. The text-mining process itself will undergo validation through iterative refinement of algorithms and checks for coherence and relevance, ensuring that semantic clusters correspond to meaningful constructs within the study’s context. This triangulation between quantitative and qualitative findings will deepen the understanding of the factors influencing operators’ well-being in HMI.

### Ethical Considerations

The study has been approved by the Ethical Committee of Human-Inspired Technologies Research Center of the University of Padova (Italy), with the protocol number (2023_212R1). Moreover, all participants will be asked to read carefully an information sheet describing all the research details, and to sign the informed consent before taking part in the study. At least one researcher will be present to answer all their questions and doubts.

## Results

At the time of publication, the data collection had already started. A first data collection session, with the involvement of 12 participants, has already been concluded. An additional session has been scheduled.

## Discussion

### Principal Findings

Previous research has already addressed many aspects of the relationship between operators and advanced production technologies, mainly focusing on job performance or work-related stress symptoms using either quantitative or qualitative methods alone. However, how the interaction between operators and advanced production technologies impacts individual subjective well-being has been neglected so far. Still, the changes in production tasks driven by technological innovation have been shown to exert negative effects on operators’ well-being [20,31]. These effects, often manifesting as increased cognitive workload, anxiety, and stress symptoms, require deeper examination [26,31,65]. Indeed, if these symptoms are not proactively addressed and prevented, they may develop into severe psychological distress, which can emerge in various forms, such as increased anxiety and depression. Over time, these symptoms can significantly impair an individual’s daily functioning, affecting their ability to work and maintain healthy interpersonal relationships. In the present cross-sectional study, we aim to address this gap by integrating quantitative and qualitative data to better comprehend the role played by several relevant workplace dynamics and factors, namely fluency in HMI and the dispositional negative attitudes toward advanced production technologies, to further promote operators’ subjective well-being in manufacturing. In this regard, from a quantitative point of view, we expect that the quality of the interaction between operators and the advanced production technologies, that is, fluency in HMI, may act as a protective factor against the development or worsening of operators’ psychological distress. Additionally, satisfaction with life, job, and career satisfaction, as well as the home-work interface, may function as independent variables that directly contribute to operators’ psychological well-being. Higher job satisfaction fosters a sense of accomplishment and professional stability, reducing vulnerability to depression, anxiety, and stress symptoms [25]. Likewise, a positive home-work interface enhances the ability to manage both personal and professional responsibilities effectively, mitigating psychological distress [24]. Moreover, satisfaction with life reflects a broader sense of contentment and well-being that buffers individuals against the cumulative effects of the workplace and life stressors [66]. Together, these variables, in conjunction with fluency, human-oriented, machine-oriented, and team-oriented, can have a positive impact on the psychological outcomes, highlighting their critical role in shaping a healthier and more sustainable working environment. Conversely, negative attitudes toward these advanced production technologies may be conceived as risk factors that could contribute to the onset of psychological distress. Given the scarcity of the scientific literature, we expect to explore operators’ lived experiences and interpret them referring to the scores at the questionnaires, thereby building a comprehensive overview of the subjective well-being of the operator who uses, interacts and collaborates with advanced production technologies. By triangulating quantitative findings with workers’ self-reported experiences and perceptions gathered through qualitative interviews, it becomes possible to explore how operators receive and adapt to the introduction of novel technologies, potentially unveiling additional sources of distress, such as fear of job loss, that may not emerge from standardized measures alone. This approach echoes previous research highlighting the value of integrating subjective perspectives to capture hidden psychosocial dynamics in technologically evolving workplaces [67].

These findings could be interpreted in light of the JD-R [16] model. Specifically, in our study, fluency in HMI appears to function as a job resource, thereby facilitating task execution, reducing frustration, and supporting affective well-being, while negative attitudes toward advanced production technologies act as psychological demands, potentially contributing to increased stress, anxiety, and depression symptoms. This dual pattern supports the JD-R perspective and underscores the importance of designing interventions that simultaneously reduce techno-stressors and enhance enabling resources in technology-intensive environments.

In this regard, as part of the study, psychoeducational interventions will be proposed to address organization-specific challenges and psychological stressors, providing operators with practical strategies to address workplace challenges and fostering a supportive organizational environment. This aligns with the principles of Industry 5.0 by placing operators’ well-being and workplace challenges at the center of interventions aimed at fostering sustainable HMI.

The multimethod approach adopted in this study further offers significant value by combining the strengths of both quantitative and qualitative data collection techniques. While the quantitative data provide a structured and systematic analysis of the relationships between cognitive well-being, affective well-being, and the mediating factors of fluency and negative attitudes toward advanced production technologies, the qualitative interviews offer an opportunity to explore these phenomena in greater depth by contextualizing the findings from the self-reported questionnaires. This sequential approach allows the application of the quantitative results to inform the focus of the interviews, thereby ensuring that the qualitative data collection is targeted. The interviews also provide nuanced insights into the subjective perspectives of operators, capturing details that are often difficult to quantify, such as their perceptions of collaboration with technologies, the organizational challenges they face, and the interpersonal and social dynamics within their workplaces. These insights complement the quantitative findings by highlighting the mechanisms through which fluency and attitudes toward advanced production technologies influence subjective well-being outcomes, as well as identifying potential areas for support that may not emerge from survey data alone. This integration of methods is particularly important in the context of Industry 5.0, where a human-centric approach reflects a comprehensive understanding of operators’ experiences to design interventions that promote well-being. The integration and triangulation of quantitative and qualitative findings, also through the use of the text mining technique, enhances the study’s methodological robustness. Addressing these issues is crucial not only for the well-being of individual operators but also for the overall health of the organization. Therefore, proactive measures will benefit both individual operators and the organization as a whole, leading to a more sustainable and productive work environment. Indeed, by fostering a work environment that addresses subjective well-being [66] intended as affective (ie, depression, anxiety, and stress symptoms) and cognitive well-being (ie, satisfaction with life, job and career satisfaction, and home-work interface) of operators, organizations can create a more resilient, engaged, and ultimately effective workforce capable of proactively adapting to the evolving demands of the modern industrial age. This conceptualization is, in fact, based on the 3 founding pillars of Industry 5.0, recognizing that the operators’ psychological well-being and optimal workplace dynamics are integral parts of sustainable productivity.

### Limitations and Future Implications

Still, we acknowledge some limitations of this study. First, it was designed to be cross-sectional, thus failing to capture the interaction between operators and advanced production technologies over time. A longitudinal design would provide an understanding of the temporal dynamics and causal relationships between cognitive and affective well-being, fluency, and negative attitudes toward advanced production technologies. However, this study adopts a cross-sectional design for several practical and methodological reasons. Conducting research across several manufacturing companies presents logistical challenges, particularly in ensuring consistent participation over time and varied work schedules typical of industrial environments [68]. Furthermore, as an exploratory study addressing, a cross-sectional design enables the establishment of baseline relationships and the potential identification of key mediating factors, laying the groundwork for future research. Second, the sample is drawn exclusively from manufacturing companies located in Northeastern Italy. While this region is a hub for industrial innovation and advanced production technology adoption, the findings may not be directly generalized to other geographical areas or industrial sectors. Nevertheless, given the ecological and human-centered nature of the current approach, we believe that the findings may retain a degree of transferability to similar manufacturing contexts, especially where similar technological transformations and psychosocial dynamics are present. Still, we have to acknowledge that differences in cultural, organizational, and technological contexts may influence the applicability of the results. Future research should expand to include diverse geographical locations and industries to validate and extend the findings that will emerge from this study. Moreover, the organizational level and all the related aspects (eg, organizational culture), which may play an important role, were not considered, as the study is specifically focused on the individual level. Additionally, certain relevant aspects, such as the fear of job loss, which may significantly impact workers’ subjective well-being, will not be assessed quantitatively in this study. Instead, this construct will be explored qualitatively through semistructured interviews, as this method allows for a more in-depth understanding of how such fears manifest in relation to specific organizational, technological, and relational dynamics. Given the exploratory nature of this study and the complexity of how fear of job loss may be experienced in the context of technological transformation, a qualitative approach is considered more suitable at this stage.

Furthermore, the voluntary nature of interview participation, as ethically required in all human-subjects research, may introduce a degree of self-selection bias. It is possible that participants with more pronounced experiences, positive or negative, were more likely to take part. Nonetheless, the qualitative data will be collected in a real-world manufacturing environment, characterized by active production shifts and organizational constraints. This ecological setting, while it naturally has its limitations, contributes to the applied value of the findings and strengthens their relevance for practical implementation. Finally, despite reasoning within the paradigm of Industry 5.0, especially emphasizing the need to bring operators to the forefront, social inclusiveness, which may be involved in their well-being, was not considered. Notwithstanding this, the investigation of the relationship between all the abovementioned variables will provide a comprehensive view of the factors that contribute to operators’ subjective well-being in HMI in manufacturing 5.0. This understanding will allow us to achieve the final goal of developing a consensus report that will provide guidance to companies that have adopted advanced production technologies, further considering the importance of the interaction between operators and machines, to improve workplace conditions and to identify adaptive organizational and clinical strategies to support operators’ well-being and optimize HMI. The consensus report will also serve as a practical framework for guiding workplace policies, such as implementing measures to foster subjective well-being and effective HMI, while addressing job satisfaction. Additionally, it will inform the development of tailored training programs for operators, ensuring they are equipped with the necessary skills to adapt to evolving technologies while maintaining a healthy work-life balance. These practical implications highlight the report’s potential to create more sustainable and operator-focused workplaces, in line with Industry 5.0 principles. Early recognition and intervention are essential to mitigating potential psychological distress and promoting operators’ mental well-being. By identifying key variables that influence subjective well-being, organizations can develop targeted psychoeducational initiatives, including training programs focused on the use, interaction, and collaboration with advanced production technologies, as well as awareness campaigns and workshops designed to foster resilience and adaptability in the workforce. In turn, this approach can improve the quality of HMI.

### Conclusions

As noted by Ghisleri and colleagues [69], the role of work in one’s life is pivotal, because it not only responds to instrumental needs, but rather it also answers to crucial intrinsic needs of maintaining self-esteem and satisfactory social relationships. Therefore, investigating whether and how new technological working tools can be effectively integrated not only in the work practices but also in the workers’ psychosocial workspace is crucial to ensure both the workers’ psychological well-being and the organization's performance. This study is not a mere statistical exploration; it aims to be an essential tool to understand and guide the complexities of contemporary workplaces, where humans and machines can coexist and should coexist harmoniously.

## Appendix

Multimedia Appendix 1 Checklist flow of the study’s methodology.

## Abbreviations

DSM-IV-TR: Diagnostic and Statistical Manual of Mental Disorders (Fourth Edition, Text Revision)
EPICOS: Exploration of Psychological Impacts in Collaborative Operator Settings
GAD-7: Generalized Anxiety Disorder-7
HMI: Human-Machine Interaction
JD-R: Job Demands-Resources
NARS: Negative Attitude toward Robots Scale
WRQoL: work-related quality of life

## Data Availability

Data sharing is not applicable to this article as no datasets were generated or analyzed during this study.
