# Supplementary figures and images for: Exploring Subjective Well-Being in Human-Machine Interaction: Protocol for a Mixed Methods, Cross-Sectional Analysis in Manufacturing 5.0
Source: JMIR Res Protoc. 2025 Nov 14;14:e73896. doi: 10.2196/73896 (PMC12663708; doi:10.2196/73896)

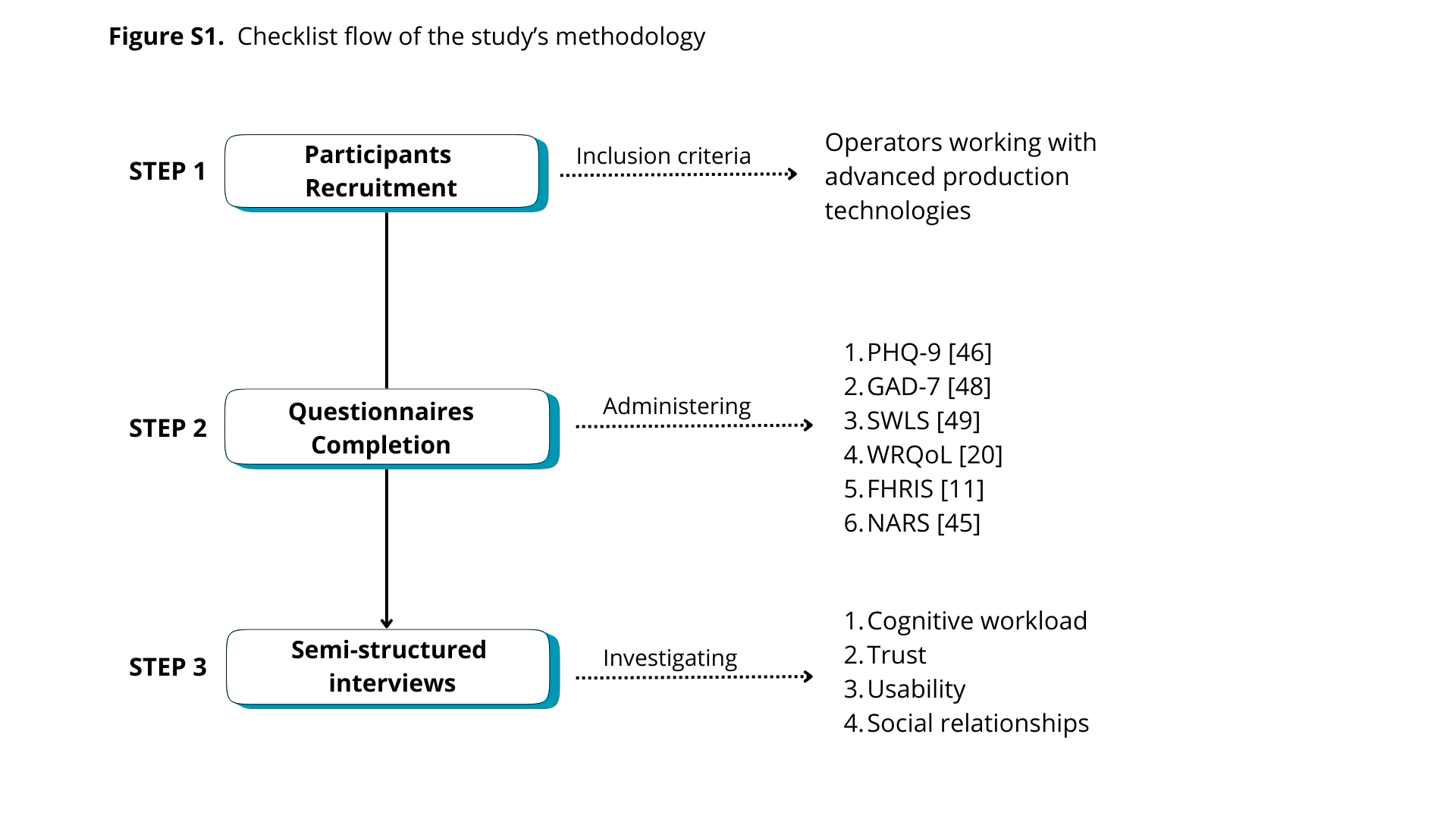

Supplement: Multimedia Appendix 1 [file resprot_v14i1e73896_app1.png]
